# HDACi and Nrf2: not from alpha to omega but from acetylation to OA

**DOI:** 10.1186/s13075-015-0885-x

**Published:** 2015-12-18

**Authors:** Holger Jahr

**Affiliations:** Department of Orthopaedic Surgery, University Hospital RWTH Aachen, Pauwelsstraβe 30, 52074 Aachen, Germany

## Abstract

Nuclear factor (erythroid-derived 2)-like 2 (Nrf2) is probably the most important ubiquitously expressed little protein that you have never heard of. Discovered more than 20 years ago, it is now known as a master regulator of redox homeostasis, controlling a plethora of cytoprotective phase II anti-oxidant enzymes. Regulation of gene expression by histone acetyltransferases and histone deacetylases drives the etiology of many age-related human diseases, including osteoarthritis. In *Arthritis Research & Therapy*, Cai et al. explore systemic histone deacetylase inhibition as a strategy to prevent osteoarthritis and identify a role for Nrf2 in preventing cartilage degeneration.

In *Arthritis Research & Therapy*, Cai et al. used a broad-spectrum histone deacetylase (HDAC) inhibitor (HDACi) and compared histological cartilage degradation between wild-type and Nrf2 (nuclear factor (erythroid-derived 2)-like 2) knockout mice, as well as expression of matrix metalloproteinases (MMPs), to conclude that acetylation-activated Nrf2 mediates HDACi protection against osteoarthritis (OA) [1].

Although certain OA susceptibility loci predict the risk for disease development, environmental parameters determine the individual disease progression. Epigenetic modifications are a mitotically heritable means by which cells can regulate gene expression, often in response to environmental changes. Actual gene expression in a cell relies on a complex interplay between the general accessibility of a coding region on the basis of the structural packaging status of the chromatin and the activation of the gene promoter by transcription (co-)factors. Basic mechanisms of epigenetic regulation are non-coding RNAs, DNA methylation, and histone variants and their modifications [2]. These post-translational histone modifications, comprising acetylation, phosphorylation, methylation, and ubiquitination [3], can act in a concerted fashion to regulate the genome accessibility, also referred to as the histone code. Acetylation occurs on specific lysine residues on the N-terminal tails of histones, mediated by histone acetyltransferases (HATs), which loosen the histone–DNA interaction, allowing access to the transcriptional apparatus in the euchromatin. In contrast, HDACs (EC 3.5.1.98) remove these acetyl groups to condense DNA into heterochromatin. Although that appears to be their predominant function, regarding HDACs solely in the context of regulating gene transcription by modifying histones and chromatin structure is too simple. HDACs are interacting with a variety of non-histone proteins; some of these are transcription factors and co-regulators, some are not. Although our knowledge about the role of DNA methylation in OA is increasing [4], effects of the histone code remain largely enigmatic. Nonetheless, age-related loss of normal epigenetic patterns is associated with numerous human diseases.

Nrf2 is a “thermostat” within our cells that senses the level of cellular stress and turns on internal protective mechanisms to orchestrate cellular defense; Nrf2 is controlled by its adaptor protein Keap1 (Kelch-like ECH-associated protein 1), which regulates its proteasomal degradation. Because Nrf2 is involved in several degenerative diseases in multiple organs [5], biogerontologists proposed that exposing cells to mild stress should result in a beneficial adaptive hermetic response. In fact, Nrf2 activation developed an imago as potential cure for all age-related diseases. Very potent Nrf2 activators were found in many cruciferous vegetables like cabbage, displaying anti-inflammatory and anti-oxidant activities [6]. Whereas the Medical School of Warwick University (Coventry, UK) started developing Nrf2 activation-based superfoods for healthier aging, a German university is evaluating traditional cabbage leaf cataplasms for treating primary symptomatic knee OA. However, a currently emerging “dark side” indicates that Nrf2 may in a context-dependent manner even promote diseases [5], which may result from divergent responses to intrinsic and extrinsic cellular stress. So, eat your veggies, but keep in mind that superfood is unlikely to become our first-line treatment for OA.

Cai et al. used trichostatin A (TSA) as an HDACi and this choice has important limitations because TSA has poor pharmacokinetics and does not discriminate between HDAC isozymes [7]. Recent data further demonstrate a crosstalk between nuclear factor-kappa-B (NF-κB) and Nrf2 in the inflammatory pathway. The p65 subunit of NF-κB is itself controlled by acetylation and deacetylation (via HDAC3 and HDAC6), and NF-κB modulates expression of MMP-1, MMP-3, and MMP-13 in cytokine-stimulated cells. MMP-1 and MMP-13, being able to degrade native cartilage-specific type II collagen, are critical factors of OA progression, but MMPs are also zinc-dependent endopeptidases and may be directly inhibited by the zinc-chelator TSA. TSA can further induce partial relaxation of genomic imprinting and decrease DNA methylation, while demethylation of the MMP-13 promoter in chondrocytes regulates its expression [8]. Next to MMPs, HDACs also transcriptionally regulate other important matrix-degrading enzymes, like key aggrecanases [9], contributing to the therapeutic potential of HDAC inhibition for arthritic diseases.

So, does acetylation-mediated Nrf2 activation exert this OA-protective effect? The evidence presented by Cai et al. does point in that direction. Also, monosodium iodoacetate-induced OA involves reactive oxygen species in chondrocytes and HDAC inhibition suppresses synovial inflammation. Heme oxygenase-1, an accepted Nrf2 target gene, was further shown to mediate beneficial effects on osteoblasts and chondrocytes from patients with OA. Acetylation of Nrf2 can enhance its promoter-specific DNA binding and appears to function in concert with, and downstream of, Keap1-mediated Nrf2 ubiquitination in modulating its activity [10]. As a dynamic and reversible process, acetylation of Nrf2 is determined by the relative activities of HATs and HDACs, both of whose activities are tightly, but diversely, regulated by many signaling pathways.

Is targeting Nrf2 activity by HDAC inhibition a promising strategy to fight OA? Where Cai et al. started, others will follow. Because some HDACs, like the sirtuins, are not affected by TSA, the good news is that the number of newly developed HDACis is rapidly increasing [6] to allow more targeted approaches. Anyway, Nrf2 was possibly the most important little protein that you have never heard of—but that has now changed!

## Abbreviations

HAT: Histone acetyltransferase
HDAC: Histone deacetylase
HDACi: Histone deacetylase inhibitor
Keap1: Kelch-like ECH-associated protein 1
MMP: Matrix metalloproteinase
NF-κB: Nuclear factor-kappa-B
Nrf2: Nuclear factor (erythroid-derived 2)-like 2
OA: Osteoarthritis
TSA: Trichostatin A
